# Uterine EMG activity in the non-pregnant sow during estrous cycle

**DOI:** 10.1186/s12917-018-1495-z

**Published:** 2018-06-05

**Authors:** Malgorzata Domino, Bartosz Pawlinski, Magdalena Gajewska, Tomasz Jasinski, Maria Sady, Zdzislaw Gajewski

**Affiliations:** Department of Large Animal Diseases with Clinic, Veterinary Research Centre and Center for Biomedical Research, Faculty of Veterinary Medicine, Warsaw University of Life Sciences (WULS – SGGW), Nowoursynowska 100, 02-797 Warsaw, Poland

**Keywords:** EMG activity, Direction, Speed, Estrous cycle, Sow, Myometrium

## Abstract

**Background:**

Uterine myoactivity is crucial for successful reproductive performance of the sow. Spontaneous contractions of the uterus are strictly controlled and coordinated**.** Uterine electromyographic (EMG) activity undergoes hormonal regulation with rapid and long-term effects. What is more, interstitial Cajal-like Cells (ICLC) appear essential for smooth muscle contractility in the reproductive tract where they are suspected to be playing a major role in generating, coordinating, modulating and synchronizing slow triggering waves. The aim of this study was to investigate the myoelectrical activity of sow’s uterus during estrus cycle.

**Results:**

Study was conducted on 10 Polish Landrace sows. Propagation mechanisms and their connection with the uterine EMG activity were considered in correlation with expression of c-kit, progesterone and oxytocin receptors of the non-pregnant sow. ICLC were labeled with antibody directed against c-kit receptor and visualized by confocal microscopy and scanning cytometer for positive cells percentage assessment. EMG signal was recorded directly from the myometrium with telemetry transmitters and electrodes located in different topographic regions of reproductive tracts*.* The stages of estrus cycle were determined by monitoring levels of luteinizing hormone, progesterone and estrogen with radioimmunoassays. Significant differences of the EMG signal parameters between diestrus and estrus and the correlations with density of labelled receptors were demonstrated. Moreover, the electrophysiological studies indicated that ICLC in the myometrium in the tip of uterine horn may participate in the regulation of slow waves duration and frequency.

**Conclusions:**

The pattern of EMG signal propagation in the wall of the non-pregnant porcine uterus occurs in an orderly, bidirectional fashion and at distinctive speed, with no differences between diestrus and estrus.

## Background

Reproductive performance of the sow is a critical component of profitable production. Spontaneous contractions of uterus must be controlled and coordinated for the success of various reproductive functions. Suitable uterine contractility is involved in the transport of gametes and embryo implantation. Unsuitable uterine contractility may lead to ectopic pregnancies, miscarriages, embryonic loss and abnormalities of puerperium.

Uterine electromyographic (EMG) activity comes under hormonal regulation due to acute and long-term effects. The acute effects promoting uterine contraction and relaxation are based on a number of intracellular molecular processes (i.e. rise in intracellular calcium_;_ increase in myosin phosphorylation; increase in myosin light-chain kinase activity) [1]. In myometrium, an increase in myosine phosphorylation accompanies spontaneous and hormone-induced contractions. Spontaneous and agonist-induced relaxation is accompanied by decrease in myosin light-chain phosphorylation [2]. The long-term effects concern hormonal regulation (including reproductive hormones) of components of intracellular systems at the level of the plasma membrane (i.e. receptor-operated, voltage-operated, second-messenger-operated, GTP-binding protein (Guanosine-5′-triphosphate-binding protein), gated ion channels) [1].

It is generally accepted that progesterone, estrogens and oxytocin are key regulators of uterine contraction [3, 4]. Progesterone promotes sustained myometrial relaxation, estrogens and oxytocin favor myometrial contractility and excitability [5]. Recent studies have suggested that contractility in spontaneously active organs occurs as an intrinsic property of the muscle [6]. At the plasma membrane level, progesterone interacts with membrane-associated receptors (progesterone receptors - PR) to directly modulate intracellular calcium and cyclic adenosine monophosphate levels. Progesterone also indirectly inhibits estrogen-induced oxytocin receptors (OXTR) expression. At the plasma membrane level oxytocin also interacts with membrane-associated receptors and induces inositol triphosphate production and Ca2+ mobilization [5].

Based on their gastrointestinal (GI) tract investigations, Torihashi et al. (1999) have proposed a population of independent interstitial cells as pacemakers of contractile rhythm.

In GI the interstitial cells of Cajal (ICC) create a network which initiates and propagates the slow waves. Moreover, cells with morphology and antigenicity similar to ICC have been found outside the gastrointestinal tract and named interstitial Cajal-like cells (ICLC). The ICLC appear essential for smooth muscle contractility in the urinary and reproductive tract where they generate, coordinate, modulate and synchronize slow triggering waves [7]. The discovery of the c-kit receptor (type III tyrosine kinase receptor; CD117) as a marker of ICLC allows for the recognition of these cells under light and confocal microscopes [8]. The presence of ICLC located among smooth muscle cells (SMC) were demonstrated in non-pregnant human [9] and porcine [10] myometrium. Moreover, the presence of estrogen, progesterone and oxytocin receptors was demonstrated in the nuclei of ICLC in human myometrium [11, 12].

Anatomical structure of uterus with billions of SMC comprising myometrium interacting in a complex manner in longitudinal and circular muscle layers intertwined with the network of pacemaker cells (ICLC) must be considered in myoactivity signal propagation analysis. Individual electrical activities within the myometrial tissue may differ in speed and direction. A single electrical activity (burst) can initiate a myometrial contraction but multiple, coordinated activities (bungle) are needed for powerful and sustained contractions [1, 13]. SMC contract when the action potentials reaches a depolarization threshold and generates an electromagnetic field, possible to measure as voltage. Therefore, electromyography (EMG) is the most accurate method to measure action potential changes leading to synchronous contraction on the organ level [14].

The primary objective of this paper is to discuss the reproductive phenomenon, which is the myoelectrical activity of sow uterus during estrus cycle. We considered physiological modeling of uterine electrical activity generated at cellular and organ level, propagation mechanisms and their correlation with the uterine EMG signal recorded internally from the myometrium and expression of c-kit, PR and OXTR of the non-pregnant sow. Although a precise role for myometrial Cajal-like interstitial cells has not yet been identified, this study presents results, that may support understanding of functional role of ICLC in regulating uterine contractile activity.

## Methods

### Animals

The experiment has been conducted on 10 mature Polish Landrace sows (*n* = 10) according to protocol approved by the III Local Ethical Committee on Animal Testing in Warsaw (Permit Number: 71/2009, from 19.11.2009) on behalf of the National Ethical Committees on Animal Testing. Spontaneous uterine activity in non-pregnant state was recorded by the combination of three electrodes connected to 3-channel transmitter used in large animals [15]. The experiment started with surgery in the diestrus and the estrus began on average between day 7 and 12 after surgery when the highest quality EMG signal was collected. One registration period lasted 5 to 6 weeks with 1.87 ± 0.40 estrus cycles occurring during this time. Surgery was carried out under general anesthesia and the telemetry EMG recording method was performed. According to the standard protocol [16] animals were premedicated with an intramuscular injection of azaperone (Stresnil, 3 [mg/kg b.wt.], IM, Janssen Pharmaceutica) and then catheter was inserted into the auricular vein. General anesthesia was achieved with combined administration of medetomidine (Cepetor, 1 [mg/kg b.wt.], IV, CP- Pharma Handelsges), butorphanol (Butomidor, 0,2 [mg/kgb.wt.], IV, Ricgter Pharma AG), ketamine (Bioketan, 3[mg/kg b.wt.], IV, Vetoquinol Biowet) and propofol (Propofol, 2 ± 4 [mg/kg b.wt.], IV, Pfizer). The telemetry transmitter TL10M3-D70-EEE (DSI, USA) was surgically positioned between abdominal muscles and electrodes sutured into different topographic regions of reproductive tract. In experiment 1: right uterine horn (RUH-channel 1), corpus uteri (CU-channel 2) and the left uterine horn (LUH-channel 3) surfaces with 17 cm distance between electrodes and in experiment 2: isthmus of oviducts (IO-channel 1), right uterine horn tip (RUHT-channel 2), right uterine horn (RUH-channel 3) surfaces with 17 cm distance between electrodes 2 and 3. When pigs recovered from surgery analgesic - meloxicam (Metacam 0.4 [mg/kg BWT], IM, Boehringer Ingelheim) and anti-microbial - cefquinom (Cobactan, 2.0 [mg/kg BWT], IM, Intervet) had been administered for 5 days. Directly after surgery, one day before the start of registration, the EMG signal quality was inspected. At day 3 to 5 after surgery the disturbance-free signal was obtained and the regular registrations was carried during next 5 to 6 weeks. The EMG signals were collected 10 h daily always in the same time periods. Obtained analog signal was digitalized and sent to the telemetric receiver (DL10 (DSI, USA)). The signal was acquired with a 3-channel transmitter PowerLab (ADInstruments, Australia) with sampling frequency 100 Hz and archived for off-line analysis. Pigs were euthanized at the end of the experiment, by Sodium Pentobarbital (Morbital 100.0 [mg/kg BWT], IV, Biowet Pulawy) in different stages of estrus cycle (half in estrus (*n* = 5) and half in diestrus (n = 5)). Full-thickness tissue samples of muscle layer were obtained from uterus (corpus-CU, middle of the horn-UH, horn tip-UHT) and oviducts (isthmus-IO, infundibulum-IFO) of all pigs (*n* = 10).

### EMG data analysis

The EMG signals were digitally filtered with a band-pass filter [5–50 Hz]. Mean and linear trends were removed [17]. Uterine contractions were defined as series of electrical potentials with amplitude above 5 μV and a duration longer than 3 s, separated from each other by the time period no shorter than 5 s. Any new electrical activity after 5 s was interpreted as a subsequent contraction [18]. Contraction was described using burst and bungle parameters. The EMG signal spectral content was analyzed in time and frequency domain features. In time domain, the bursts represented single action potentials, while the bungles comprised of multiple action potentials. Mean amplitude [mV], mean RMS (root mean square) [mV], duration of electrical activity [s], duration of pauses [s], and number of bursts forming a bungle were analyzed. In frequency domain, means of Fourier analysis (FFT–Fast Fourier transform), dominant frequency (DF) [Hz] (the frequency at which most signal energy was transmitted) was assessed for each data series. We used the Hamming window for the Fourier analysis [19, 20]. The similarity measures were used to describe synchronization between EMG signals. Degree of synchronization between three simultaneously recorded data series (channels 1(x), 2(y), 3(z) in experiment 1) was estimated for two signal pairs (xy and yz) using linear measures: the cross-correlation function (ƒx,y(l), ƒy,z(l)) and the cross-coherence function (Cxy(ƒ), Cyz(ƒ)) [19, 21]. Similarity measures, based on the concept of time series data synchronization, allows signal propagation detecting in different topographic regions. It was used to “identify” the frequency fingerprint of the spatial bungle and determine, if the bungle reached the other location during propagation. Locations of the first and second electrodes (on the x/y or y/z axis) were used to measure highly synchronized signals, and the direction of bungle propagation was evaluated. The second signal location in the time function pointed to cervico-tubal and tubo-cervical directions. The speed of bungle propagation was evaluated basing on the time elapsed for highly synchronized signals to move from the first electrode to the second one.

### Microscopy analysis

Samples were fixed in paraformaldehyde and embedded in paraffin using standard histology procedures. Afterwards, samples were cut to 5 μm thin sections and stained with hematoxilin-eosine (H-E) using a standard protocol. Immunofluorescent labeling against CD117 (c-kit), progesterone receptor (PR) and oxytocine receptor (OXTR) was performed. For the assessment of immunofluorescent reaction (CD117) specificity, porcine ileum paraffin blocks were used as positive control (PC), while staining protocols, with the primary antibody omitted, were used as negative control (NC).

Immunofluorescent labeling against CD117 (c-kit), PR and OXTR was performed using a published staining protocol [8, 15]. Antigens were retrieved through citrate buffer heating, washing with PBS-buffer and blocked with BSA to minimize non-specific antibody binding. Slides were then incubated with antibodies: anti-CD117 (goat polyclonal anti-human CD117; RD System, USA; AF332; 120 min, room temperature; dilution 1:200), anti-PR (mouse monoclonal anti-human Progesterone Receptor 1A6; PROGEN, Germany, no16077; 120 min, room temperature; dilution 1:250), anti-OXTR (mouse monoclonal anti-human hOXTR; R&D Systems, USA, MAB6616; 180 min, room temperature; concentration 15 μg/ml). Cross-reactivity for above-mentioned antibodies has been confirmed in previous studies [15, 22]. Polyclonal Alexa Fluor 405 (chicken anti-mouse), Alexa Fluor 488 (chicken anti-mouse, chicken anti-goat), Alexa Fluor 660 (chicken anti-mouse, chicken anti-goat) (Abcam, UK) labeled antibodies (dilution 1:500, 120 min, room temperature) were used respectively to detect the primary antibodies. Nuclei were counterstained with 7-Aminoactinomycin D (Sigma Aldrich, Poland) or HOECHST33342 (Sigma Aldrich, Poland). After labeling, the coverslips were mounted using mounting medium for fluorescence microscopy. Immunofluorescence labeled cells were examined with confocal microscope (FV-500, Olympus, Poland). Quantitative evaluation was performed using a scanning cytometer (SCAN^R, Olympus, Poland, magnification × 200). The positive cells density was defined as a number of cells with appropriate immunophenotype and morphology in 20 fields of view (mean density ± SD). Afterwards the relative density (%) was calculated as percentage of positive cells relative to the highest density region.

### Hormone concentrations analysis

During the surgery, a silicone cannula was inserted into the brachial vein to enable collection of blood samples. Each day during the experiment (from 7 days before to 42 days after surgery) 7 mL sample of blood was collected every 4 h. All samples were used to determine stages of estrus based on luteinizing hormone (LH), 17β-estradiol (E2) and pregn-4-ene3,20-dione (P4) concentrations. The serum LH concentrations were measured with noncommercial radioimmunoassay test [21]. The sensitivity was 0.08 ng/mL (intra-assay CV < 6.7%, interassay CV < 12.5%). The serum levels of E2 and P4 were determined with RIA. The sensitivity for estradiol was 5 pg/mL (intra-assay CV < 8.0%, interassay CV < 11.1%; ESTR-US-CT; Cisbio assays, France) and for progesterone 0.15 ng/mL (intra-assay CV < 5.6%, inter-assay CV < 8.8%; KIP1458; DIAsource ImmunoAssays SA, Belgium). The stages of estrus cycle were determined by monitoring hormone levels and divided into estrus (mean concentration: E2 > 8 pg/ml, P4 < 1 ng/ml, LH > 4 ng/ml) and diestrus (mean concentration: E2 < 2 pg/ml, P4 > 4 ng/ml, LH < 1 ng/ml).

### Statistical analysis

All statistical evaluation was performed by Graph-Pad InStat software (San Diego, USA), the level of statistical significance was set to *P* < 0.05, using specific tests: Mann-Whitney test, Kruskal-Wallis test with Dunn’s multiple comparisons, one-way ANOVA test with Tukey’s multiple comparisons. The Pearson correlation coefficient (r) and Spearman’s rank correlation coefficient (Sr) were used to determine correlation between receptors (c-kit, PR, OXTR) distribution and for selected EMG parameters in different regions of reproductive tract.

## Results

The bungle parameters were characterized in details in three different topographic regions of the reproductive tract, with significant differences between diestrus and estrus (Table 1). We demonstrate significantly higher amplitude and RMS (*P* < 0.0001) and lower duration of electrical activity (P < 0.0001) in estrus compared to diestrus in all uterine regions (UHT, UH, CU). We also demonstrate significantly higher amplitude (*P* = 0.0003) and RMS (P < 0.0001) in estrus compared to diestrus in IO with no differences in duration of electrical activity. There were no differences (*P* > 0.05) in amplitude, RMS and duration of electrical activity in IO and uterus (UHT, UH, CU) in diestrus, but in estrus, amplitude and RMS were significantly higher (*P* < 0.05) and duration of electrical activity was significant lower (P < 0.05) in CU and UH compared to the oviductal region (IO, UHT).

**Table 1:** The EMG signal parameters in different topographic regions of reproductive tract during diestrus and estrus

	**EMG**		**Diestrus**		**Estrus**	
**Region**	**parameter**		mean	SEM	mean	SEM
	**A** ^**1**^	[mV]	0.64^a^	±0.04	0.81^b^	±0.02
**IO**	**RMS** ^**2**^	[mV]	0.05^c^	±0.003	0.12^d^	±0.001
	**D** ^**3**^	[s]	153.10^e^	±9.64	111.10^ef^	±14.48
	**A** ^**1**^	[mV]	0.71^a^	±0.03	0.99^b^	±0.04
**UHT**	**RMS** ^**2**^	[mV]	0.09^c^	±0.002	0.12^d^	±0.002
	**D** ^**3**^	[s]	194.70^e^	±9.25	99.97^f^	±20.97
**UH**	**A** ^**1**^	[mV]	0.74^a^	±0.02	2.22^g^	±0.15
	**RMS** ^**2**^	[mV]	0.10^c^	±0.001	0.26^h^	±0.02
	**D** ^**3**^	[s]	221.60^e^	±25.53	33.30^i^	±2.73
**CU**	**A** ^**1**^	[mV]	0.57^a^	±0.02	4.24 ^j^	±0.19
	**RMS** ^**2**^	[mV]	0.07^c^	±0.001	0.47^k^	±0.02
	**D** ^**3**^	[s]	209.70 ^e^	±22.92	42.60 ^l^	±3.23

^1^A-amplitude, ^2^RMS-root mean square, ^3^D-duration of electrical activity. The different letters in superscript indicate the statistical significant differences (*P* < 0.05): diestrus/estrus and regions of reproductive tract

The EMG signals were propagated along the UH in both cervico-tubal (CU → UH) and tubo-cervical (UH → CU) directions during diestrus and estrus with no significant differences (*P* > 0.05) (Table 2). There were no significant differences (P > 0.05) between the percentages (mean% ± SEM) of bungle propagated CU → UH during diestrus (45.30 ± 3.27) and estrus (50.40 ± 1.95) and UH → CU during diestrus (54.73 ± 3.25) and estrus (49.74 ± 1.95) in relation to the total number of highly synchronized uterine contraction signals.

**Table 2:** EMG signal propagation along uterus during diestrus and estrus

The directions of EMG signal propagation						
	LUH-CU^1^	RUH-CU^2^	UH(L,R)-CU^3^	CU-LUH^4^	CU-RUH^5^	CU-UH(L,R)^6^
Diestrus						
mean %	24.51^a^	30.20^a^	**54.73** ^b^	24.51^a^	20.82^a^	**45.30** ^b^
SEM	±2.27	±1.07	±**3.25**	±0.53	±3.75	±**3.27**
Estrus						
mean %	26.60^a^	23.13^a^	**49.74** ^b^	27,32^a^	23.11^a^	**50.40** ^b^
SEM	±1.55	±0.35	±**1.95**	±0.70	±1.15	±**1.95**

^1^LUH➔CU; ^2^RUH➔CU; ^3^UH(L,R)➔CU; ^4^CU➔LUH; ^5^CU➔RUH; ^6^CU➔UH(L,R). The different letters in superscript indicate the statistical significant differences (*P* < 0.05): diestrus/estrus and the directions of EMG signal propagation

The EMG signals propagated along the UH with three significantly different (*P* < 0.0001), independent speeds: SBMR (slow basic migration rhythm), RBMR (rapid basic migration rhythm) and RAMR (rapid accessory migration rhythm). There were no significant differences (*P* > 0.05) between EMG signal propagation speeds values along the UH and percentage of each speeds during diestrus compared to estrus (Table 3).

**Table 3:** EMG signal propagation speeds along uterus during diestrus and estrus

The speeds of EMG signal propagation			
	SBMR^1^	RBMR^2^	RAMR^3^
Diestrus			
mean speed [mm/min]	1.25^a^	2.47^b^	7.07^c^
SEM	±0.04	±0.08	±0.60
%of speeds	46.01^d^	26.30^d^	27.30^d^
Estrus			
mean speed [mm/min]	1.16^a^	2.53^b^	8.70^c^
SEM	±0.05	±0.10	±0.67
%of speeds	33.03^d^	29.01^d^	38.01^d^

^1^SBMR-slow basic migration rhythm; ^2^RBMR-rapid basic migration rhythm; ^3^RAMR-rapid accessory migration rhythm. The different letters in superscript indicate the statistical significant differences (*P* < 0.05): diestrus/estrus and the speeds of EMG signal propagation

**Fig. 1:**
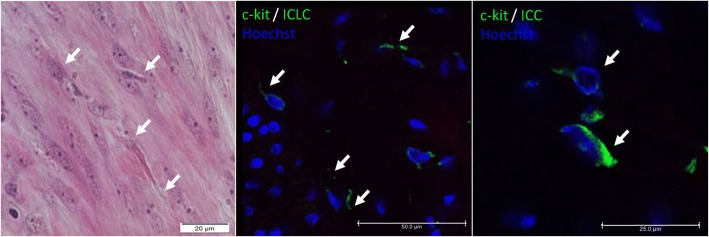
The representative images of presence of Cajal cells in uterine tissues (ICLC) and ileum (ICC). Fusiform cells with long processes and oval nucleus show morphology typical for Cajal cells (arrows). Legend: A. Muscular layer of UHT. Widefield microscopy of a H-E stained tissue section, lens magnification 40×; B. Muscular layer of UHT. Confocal microscopy following IF labeling, lens magnification 40×; C. Muscular layer of porcine ileum (PC - positive control). Confocal microscopy following IF labeling, lens magnification 63×

**Fig. 2:**
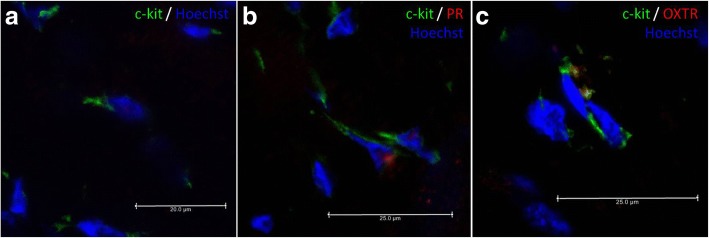
The representative images of IF labelling of c-kit (CD117), PR and OXTR. Legend: A. Muscular layer of UHT - Triangular ICLC positive for c-kit only; emission spectra of AF 488/Hoechst; B. Muscular layer of UHT - Star-like shape ICLC double positive for c-kit and PR intense at nuclear level; emission spectra of AF 488/AF 660/Hoechst; C. Muscular layer of UHT - Fusiform ICLC double positive for c-kit and OXTR intense at membrane level; emission spectra of AF 488/AF 660 /Hoechst. Confocal microscopy, lens magnification 63×, digital zoom 0.75–3.00

The cells with morphology similar to ICLC were shown by H-E staining, however the reliable differentiation of m-ICLC from fibroblasts according to Hutchings et al. [12] criteria was possible in porcine specimen after immunofluorescence labeling. Therefore, the histology approach was used only for initial screening and to examine tissue architecture and the cells with antigenicity similar to ICLC were demonstrated in all in examined parts of the porcine reproductive tract. The c-Kit-immunopositive nucleated cells that present characteristic cell morphology (triangular, spindle-shaped, star-like body with two or more, very long, moniliform processes) were demonstrated in each topographic regions of the reproductive tract. The presence of similar cells was confirmed in positive control-porcine ileum used as reference material and so the c-kit positive cells were considered as ICLC (Fig. 1). The lack of such cells was demonstrated in negative control. We demonstrated the presence of ICLC that were single-positive for c-kit and double positive for c-kit/PR as well as c-kit/OXTR in all examined topographic regions. Double immunofluorescence confirmed the same distribution of PR and OXTR on c-kit positive cells: intense in nuclei and weak in the cytoplasm for PR and intense at membranes and weak in the cytoplasm for OXTR (Fig. 2).

In recent study we demonstrated that the ICLC density differed among different parts of the porcine reproductive tract during diestrus [15]. In present study we assessed corresponding differences in estrus and compared our current results with previous data in one table in order to test the dependency of density differences on stages of estrus cycle (Table 4). Current results demonstrate that in estrus there is significantly higher (*P* < 0.01) density of ICLC in the IO compared to UH with no differences (*P* > 0.05) between UHT and UH. We also demonstrate significantly lower density of ICLC in IFO (P < 0.01) and in CU (P < 0.01) when compared to IO. There were no significant differences (P > 0.05) between density of ICLC in different topographic regions of the reproductive tract during diestrus [15] and estrus (Table 4).

**Table 4:** ICLC density (mean ± SD) in topographic regions of reproductive tract during diestrus [15] and estrus

	IFO	IO	UHT	UH	CU
Diestrus					
mean density	2.16^a^	4.12^b^	3.10^c^	3.17^c^	1.22^d^
SD	±0.82	±1.01	±0.92	±1.04	±0.38
relative density	52.4%^a^	100.0%^b^	75.3%^c^	77.1%^c^	29.6%^d^
Estrus					
mean density	2.31^a^	5.35^b^	4.23^c^	3.96^c^	1.09^d^
SD	±0.03	±0.53	±0.50	±0.12	±0.68
relative density	43.1%^a^	100.0%^b^	79.0%^c^	74.0%^c^	20.4%^d^

Immunofluorescent labeling; Density defined as cells number in 20 fields of view and relative density as percentage of positive cells relative to the highest density region. The different letters in superscript indicate groups with statistically significant differences (P < 0.05): diestrus/estrus and regions of reproductive tract

The correlations (Pearson’s r or Spearman’s rank) between ICLC, PR, OXTR density (mean ± SD) and the following EMG signal parameters: dominant frequency, amplitude, RMS, duration of bungles and bursts, duration of pauses between bungles and bursts, number of bursts forming bungle (mean ± SEM) were calculated for different topographic regions of the reproductive tract during diestrus and described below.

Strong positive correlation has been confirmed in CU (*r* = 0.86; *P* = 0.03) between ICLC density (1.22 ± 0.38) and OXTR density (0.53 ± 0.02) and strong, negative correlation (Sr = − 0.71; *P* < 0.0001) between PR (0.20 ± 0.02) and number of bursts forming bungle (3.40 ± 0.40).

In UH there was strong negative correlation (Sr = − 0.87; *P* < 0.0001) between ICLC density (3.17 ± 1.04) and number of bursts forming bungle (3.40 ± 0.24); strong positive correlation (*r* = 0.82; *P* = 0.04) between PR density (0.31 ± 0.02) and EMG signal dominant frequency (4.01 ± 0.21); strong negative correlation (*r* = − 0.86; *P* = 0.03) between OXTR density (0.61 ± 0.01) and EMG signal amplitude (1.18 ± 0.17); strong positive correlation (*r* = 0.85; *P* = 0.03) between OXTR density (0.61 ± 0.01) and EMG signal dominant frequency (4.40 ± 0.31) and very strong positive correlation between OXTR density (0.61 ± 0.01) duration of bungles (3.77 ± 0.46) (*r* = 0.99; *P* = 0.01) and duration of bursts (9.21 ± 1.54) (*r* = 0.97; *P* = 0.03). Strong negative correlations between OXTR density and duration of pauses between bungles (7.23 ± 1.54) (*r* = − 0.94; P = 0.01) and duration of pauses between bursts (13.20 ± 2.73) (*r* = − 0.97; P = 0.03) were observed.

In UHT there was strong negative correlation between ICLC density (3.10 ± 0.92) and duration of bungles (2.31 ± 0.36) (*r* = − 0.85; P = 0.03) as well as duration of bursts (9.59 ± 0.88) (*r* = − 0.86; P = 0.03) and very strong positive correlation (*r* = 0.95; P = 0.01) between OXTR density (0.52 ± 0.03) and EMG signal RMS (0.11 ± 0.01).

Last but not least, in IO there was strong negative correlation between ICLC density (4.12 ± 1.01) and duration of pauses between bungles (2.72 ± 1.26) (*r* = − 0.81; *P* = 0.04) as well as between OXTR density (0.90 ± 0.02) and duration of pauses between bursts (5.08 ± 0.10) (*r* = − 0.82; P = 0.04).

## Discussion

In this study, we have thoroughly and systematically characterized the electrical activity of the porcine uterus throughout the estrus and diestrus. Our complex experiment combines parallel studies: on cellular level in situ and in vivo on organ level, in living organism, encompassed by natural biological processes. In pigs, an activity of the myometrium seems to be myogenic in origin, similarly to described in Langendijk et al. (2002) reports, and to be driven by pacemaker activity of not only the uterine smooth muscle cells [23] but also ICLC similar to these found in humans [12]. The anatomical arrangement of SMC in uterus with ICLC located as a network between SMC is consistent with the immunohistochemical observations in humans [9] and pigs [10].

Popescu et al. (2005) showed that ICLC establish close contacts (gap junctions) with each other, SMC, nerve fibers and capillaries. These authors also stated that reproductive hormones could influence ICLC function via Cx43-mediated mechanism [23, 24] and via OXTR and PR [25]. Cretoiu et al. (2009) suggested that ICLC could act as steroid hormone sensors [12, 25]. In the current manuscript we present the data showing that ICLC, OXTR and PR are functionally connected in porcine uterus and oviduct. This relation is particularly visible in CU, where significant correlations between ICLC and those receptors were demonstrated.

Furthermore, Hutchings et al. (2009) described the stimulatory function of ICLC on neighbouring SMC as an acute effect of promoting uterine contraction. They suggested that ICLC generate the subsequent calcium wave travelling at a relatively lower velocity towards the center of the smooth muscle fibers. [12]. In all porcine specimens the ICLC were seen to be located mainly on the boundaries of smooth muscle bundles throughout the myometrium and formed the cell network similar to that found in human tissues [26]. The presence of high correlations between steroid hormone receptors, ICLC and parameters of EMG signal suggests that in vivo ICLC could behave as hormone sensors controlling the uterine contraction and playing the role of major pacemaker, in a similar way to that found in humans [27]. In porcine specimens this network can be observed on structural and cellular level; moreover, it seems to play a functional role, but for this conclusion further research is necessary. The results presented in current manuscript support the functional role of ICLC in regulating uterine contractile activity.

Spontaneous myoactivity, as well as contractility of the cycling, nonpregnant uterus in pigs has been studied using nonsurgical, open-end catheter technique [28] and long-term electromyography combined with the telemetry recording system [18, 29, 30]. The induced myometrial activity during estrus in sow has also been studied due to clenbuterol, cloprostenol [28], oxytocin [4] and semen effect [31]. Similarly, to recent studies, we supposed that in porcine uterus and oviduct there are certain motility patterns that are more prevalent at each phase of the cycle. Signal parameters and its propagation depend on properties of wave propagation medium. Current report is, to our knowledge, the first to approach the problem of complex signal propagation medium, such as syncytium of myometrium [32], by parallel indirect research (in vivo EMG signal analyzing) as well as direct research (in situ cellular structure analyzing) in pigs. Significant difference in properties of the propagation medium in estrus and diestrus was reported [3]. Bower (1974) suggested, that in sows uterine contractions occur along uterine horns in two opposite directions, tubo-cervical and cervico-tubal, although the tubo-cervical direction appeared to be predominant [33]. Our results suggest that EMG signals in porcine uterus are propagated relatively uniformly in both cervico-tubal and tubo-cervical directions, with no differences between estrus and diestrus. In estrus, when EMG signal was propagating in tubo-cervical direction, there was strong trend for increase of signal energy, amplitude and duration of electrical activity. However, in cervico-tubal direction these values were rather decreasing. These tendencies were not observed in diestrus, which may suggest participation of specific modulators, dependent on hormonal status, that are controlling and connecting myometrium activity. In a recent study there was speculation about ICLC involvement in myometrial contractility regulation as steroid hormone ‘sensors’ [11, 12, 25, 34]. This is consistent with our results and the hypothesis that there may be a functional connection between ICLC and endocrine regulation of reproductive system. This hypothesis is based on structural relationships, direct contact of ICLC processes with blood vessels and nerve terminals observed in microscopic studies [23, 24] and the distribution of ER and PR [11]. Under in vitro conditions the uterine myocytes, in contrast to c-kit positive cells, were predominantly negative for ER and PR at the fourth passage [11]. It has to be noted, however, that ER and PR expression on human uterine SMC in culture significantly decreases with every passage and is much lower compared to cells in situ [34].

Recent studies have stated that the propagation of myoelectrical activity in uterus is linear and that speed of this propagation can be measured but have not presented any measurements showing the speed of EMG signal propagation along porcine reproductive tract [35, 36]. We propose to divide the propagation speed in porcine non-pregnant uterus into one of three distinctive categories: SBMR, RBMR and RAMR; there is, however, no evidence for any differences in any of those categories between estrus and diestrus.

Recent studies show that in mice [37] and rats [38] contractile activity is predominantly initiated from IO and UHT and it is the dominant peacemaking site of reproductive tract. Gajewski et al. (2001) suggested that also in pigs both UHT act as tentative anatomical regions with the strong pacemaker activity [30]. Our findings support this hypothesis in porcine specimen in estrus state. In both estrus and diestrus the highest density of ICLC was found in the IO and UHT. At the same time the EMG signal parameters were significantly “sharper” (higher amplitude and RMS with lower duration of electrical activity) in estrus compared to diestrus in oviductal region, similar to recent studies [18, 39].

According to Radhakrishanan et al. (2000), stimuli for SMC originates from ICLC, or neighboring myocytes and is affecting surrounding and distant (via processes creating network) cells organizing syncytium [40]. Frequency, amplitude and duration of contractile wave depend upon number of stimulated simultaneously cells [41]. Strong correlations stated in IO and UHT between time-dependent variables (duration and pause between bungles and bursts, as well as RMS) suggest regulating function of duration of myoelectric activity of those parts. It may be inferred from that findings that IO and UHT determines the rhythm of uterus contractility. This theory is confirmed with strong correlation between ICLC density and duration of bursts and pauses between bungles. Region IO/UHT may have a pacemaker function, however this thesis requires further investigation. In human studies Andersen and Barclay (1995) suggested that action potential may be generated in random pacemaker cells in reproductive system, however Popescu et al. (2007) stated that IO is possibly responsible for contractility stimulation in rats and humans [24, 42]. In our research we support hypothesis that analogous mechanism is functional in sows.

ICLC density in UH and CU was relatively lower than in IO and UHT, nonetheless EMG signal parameters differences between estrus stage are similar and even more expressed. Those findings are convergent with results obtained in vitro from mouse and rat specimens [36, 37]. Data obtained from rodent models suggested that contractions in UH and CU persisted following blockade of activity at the oviductal region [37]. Strong correlation between amplitude, dominant frequency and OXTR density, as well as between PR density and dominant frequency indicates possibility for strengthening or weakening signal generated in UHT during its propagation. Both force of contraction (amplitude, RMS) and contraction duration (duration and pauses between bungles and bursts) are probably regulated via receptors for reproductive system hormones. Oxytocin demonstrates higher activity than progesterone, which is consistent with biological outcome of those hormones. It is thought that effect of oxytocin on contractility is stronger than progesterone [43, 44], which is compatible with our findings.

In gastrointestinal tract frequency of slow waves in smooth muscles is regulated by ICC, while amplitude depends on neuroendocrine activity [45]. Correlations shown in our research suggest that relations in reproductive tract are very similar, what has been previously assumed [8, 11, 32, 46].

On the other hand, no significant correlations between key parameters of EMG and density of ICLC, OXTR, PR has been concluded in CU, which may suggest that this part of uterus is rather passive in regulation of myoelectric activity of reproductive system in sow. In previous studies it has been shown, that UH are more susceptible on regulation than CU [47]. However, basing on the previously published data and our results it may be concluded that spontaneous electric activity can be generated in UH and CU, independently of the oviduct end, nonetheless OI/UHT is suspected to have paramount pacemaker function.

## Conclusion

The pattern of EMG signal propagation in the wall of the non-pregnant porcine uterus is not random, but it occurs in an orderly, bidirectional fashion and at distinctive speed, with no differences between diestrus and estrus. The electrophysiological studies indicate that ICLC in UHT may participate in the regulation of slow waves of duration and frequency similar to that of ICC (Interstitial cell of Cajal) in gastrointestinal tract [48]. Myoelectric activity may be generated in various parts of reproductive tract, however UHT seems to be superior in contractility regulation.
